# Supplementary figures and images for: Ssl2/TFIIH function in transcription start site scanning by RNA polymerase II in Saccharomyces cerevisiae
Source: eLife. 2021 Oct 15;10:e71013. doi: 10.7554/eLife.71013 (PMC8589449; doi:10.7554/eLife.71013)

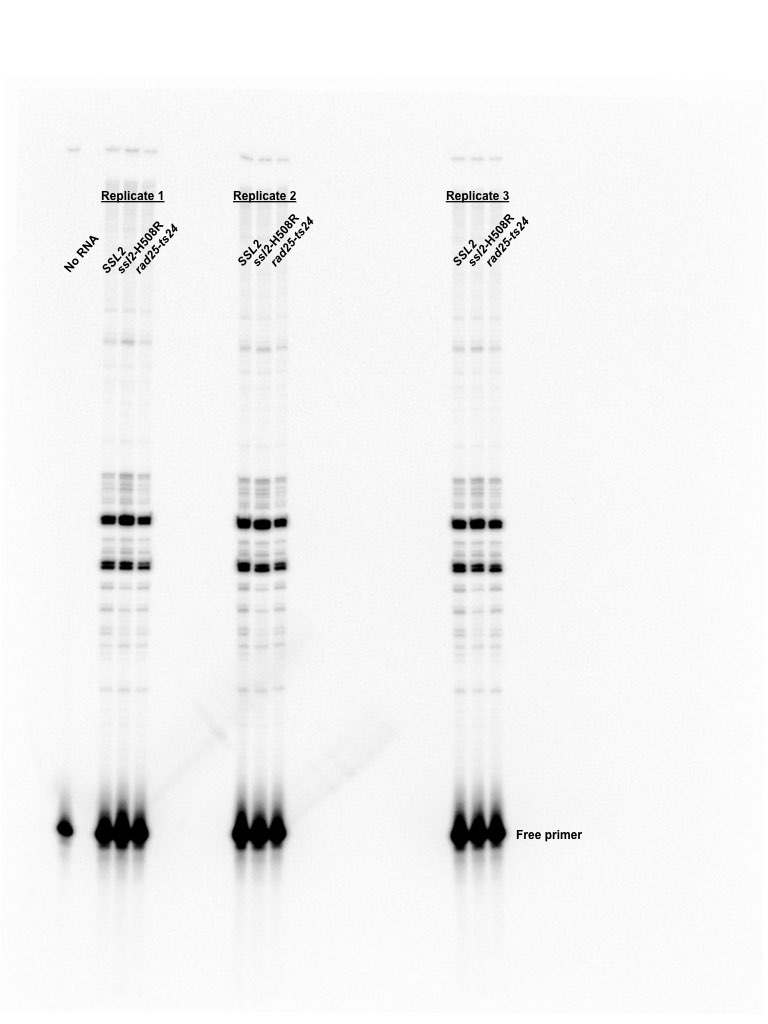

Supplement: Figure 1—source data 1. [file elife-71013-fig1-data1.zip › Figure 1-source data 1.jpeg]

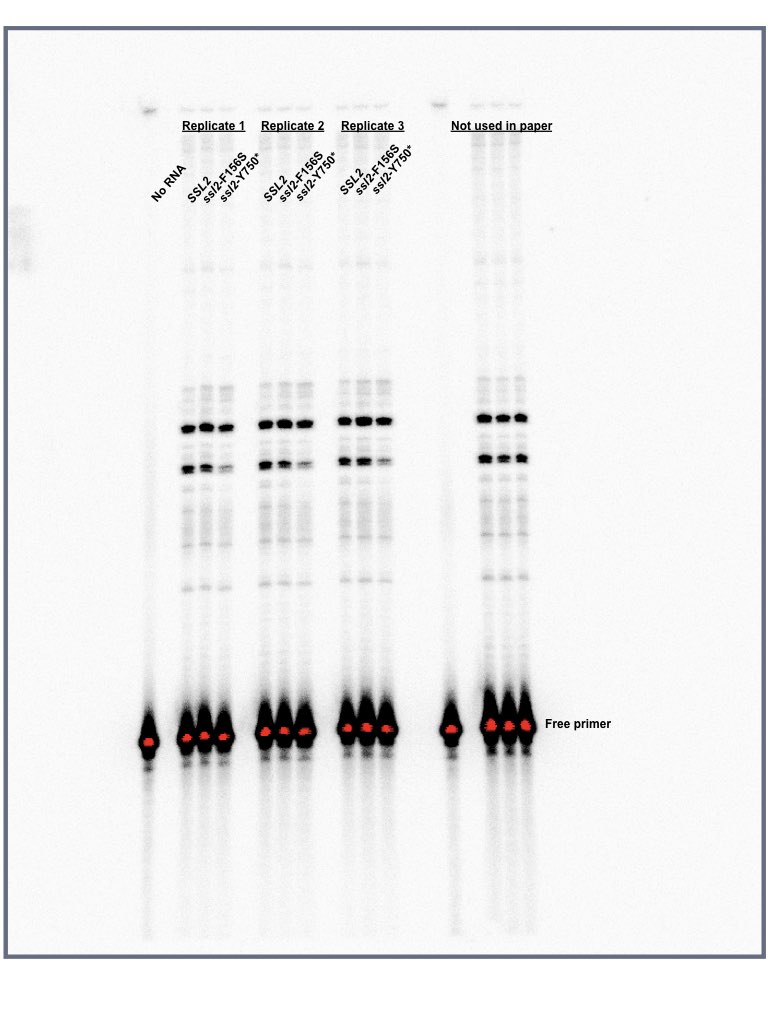

Supplement: Figure 1—source data 2. [file elife-71013-fig1-data2.zip › Figure 1-source data 2.jpeg]

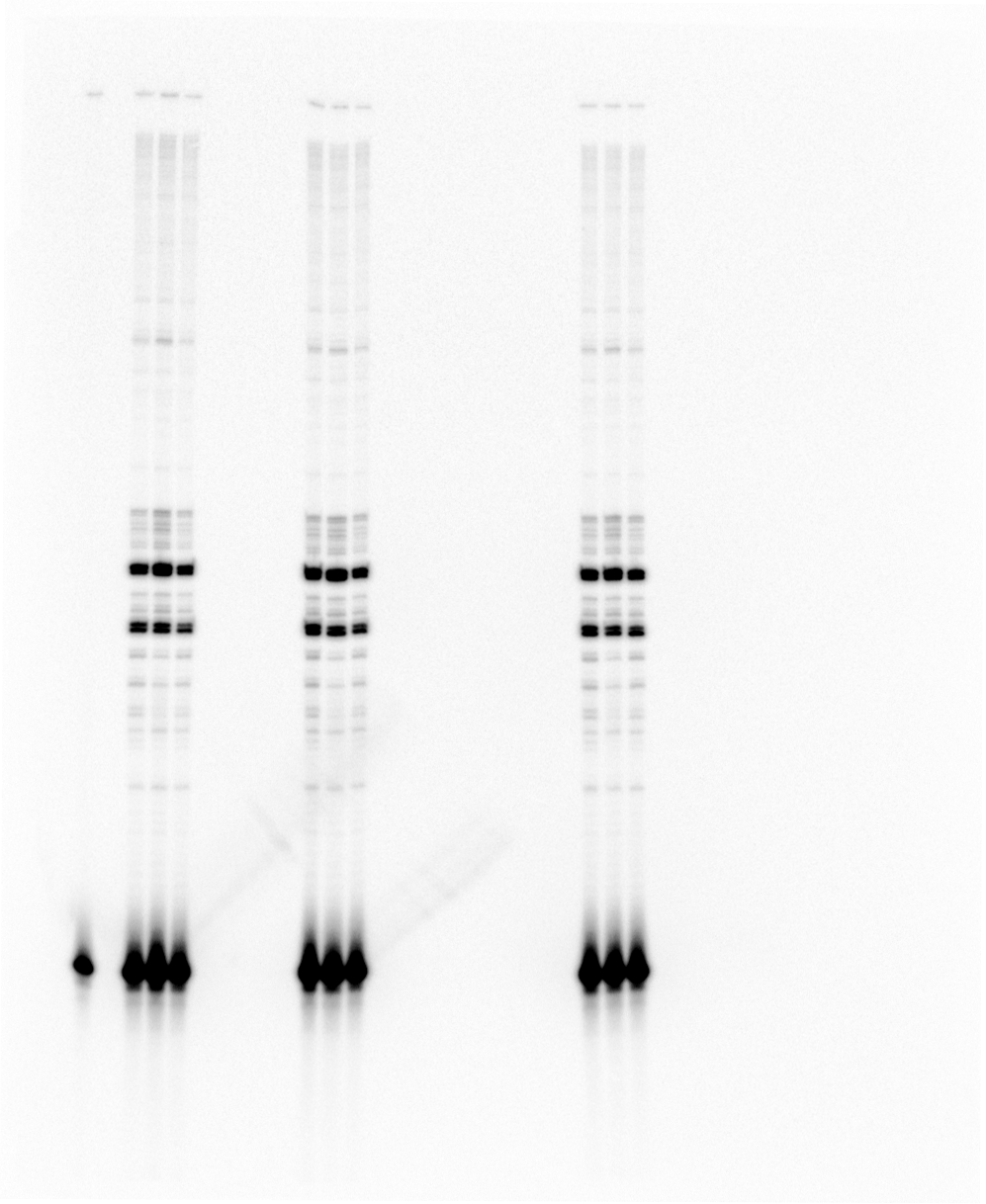

Supplement: Figure 1—source data 4. [file elife-71013-fig1-data4.zip › Figure 1-source data 4.tif]

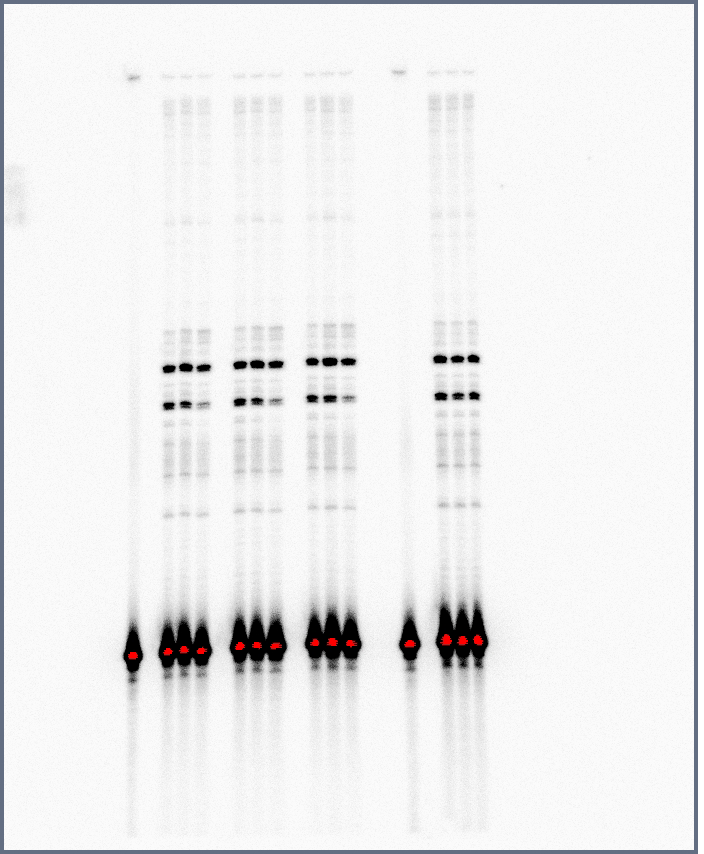

Supplement: Figure 1—source data 5. [file elife-71013-fig1-data5.zip › Figure 1-source data 5.tif]

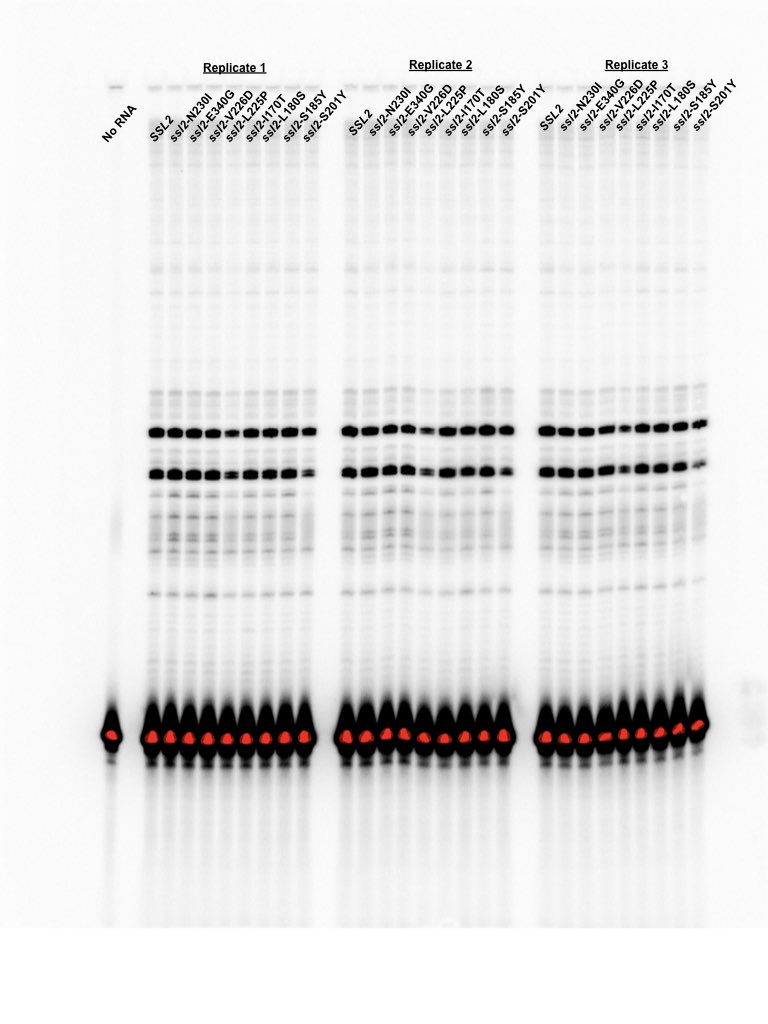

Supplement: Figure 2—figure supplement 2—source data 1. [file elife-71013-fig2-figsupp2-data1.zip › Figure 2-Figure supplement 2A source data 1.jpeg]

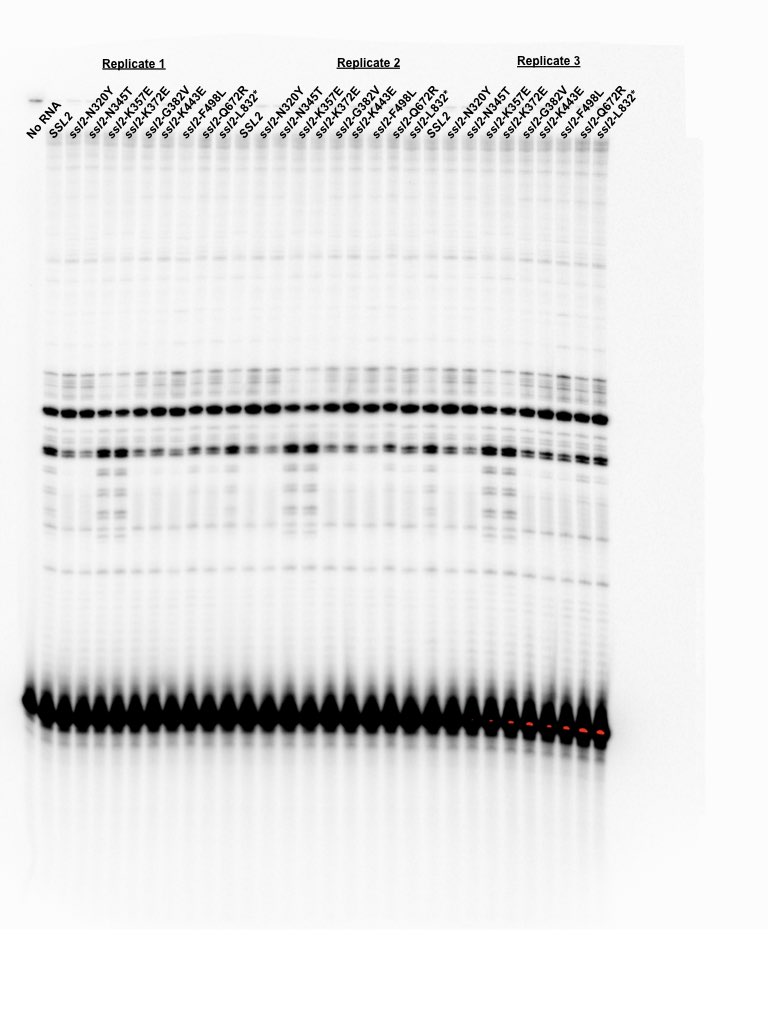

Supplement: Figure 2—figure supplement 2—source data 2. [file elife-71013-fig2-figsupp2-data2.zip › Figure 2-Figure supplement 2A source data 2.jpeg]

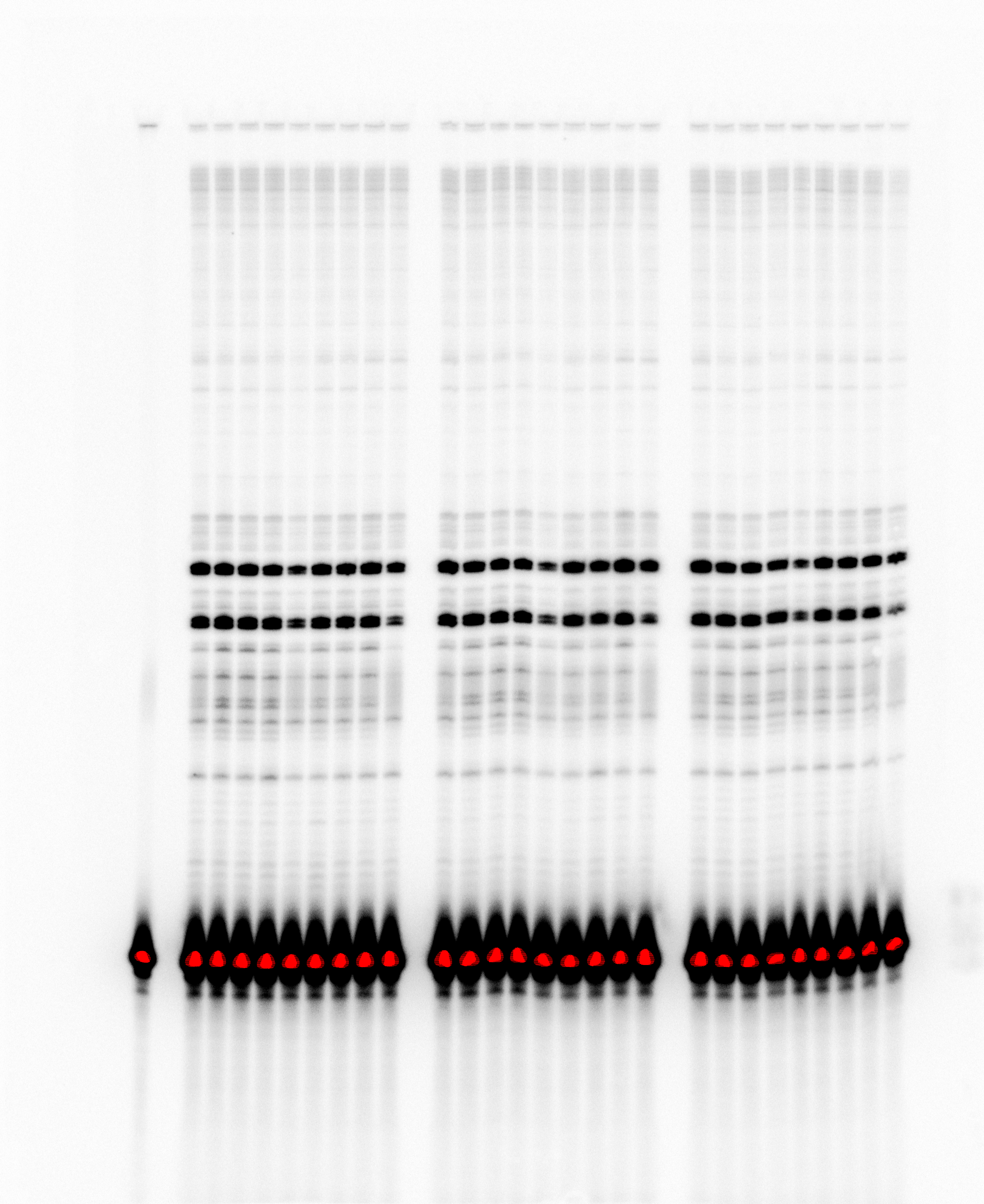

Supplement: Figure 2—figure supplement 2—source data 4. [file elife-71013-fig2-figsupp2-data4.zip › Figure 2-Figure supplement 2-source data 4.tif]

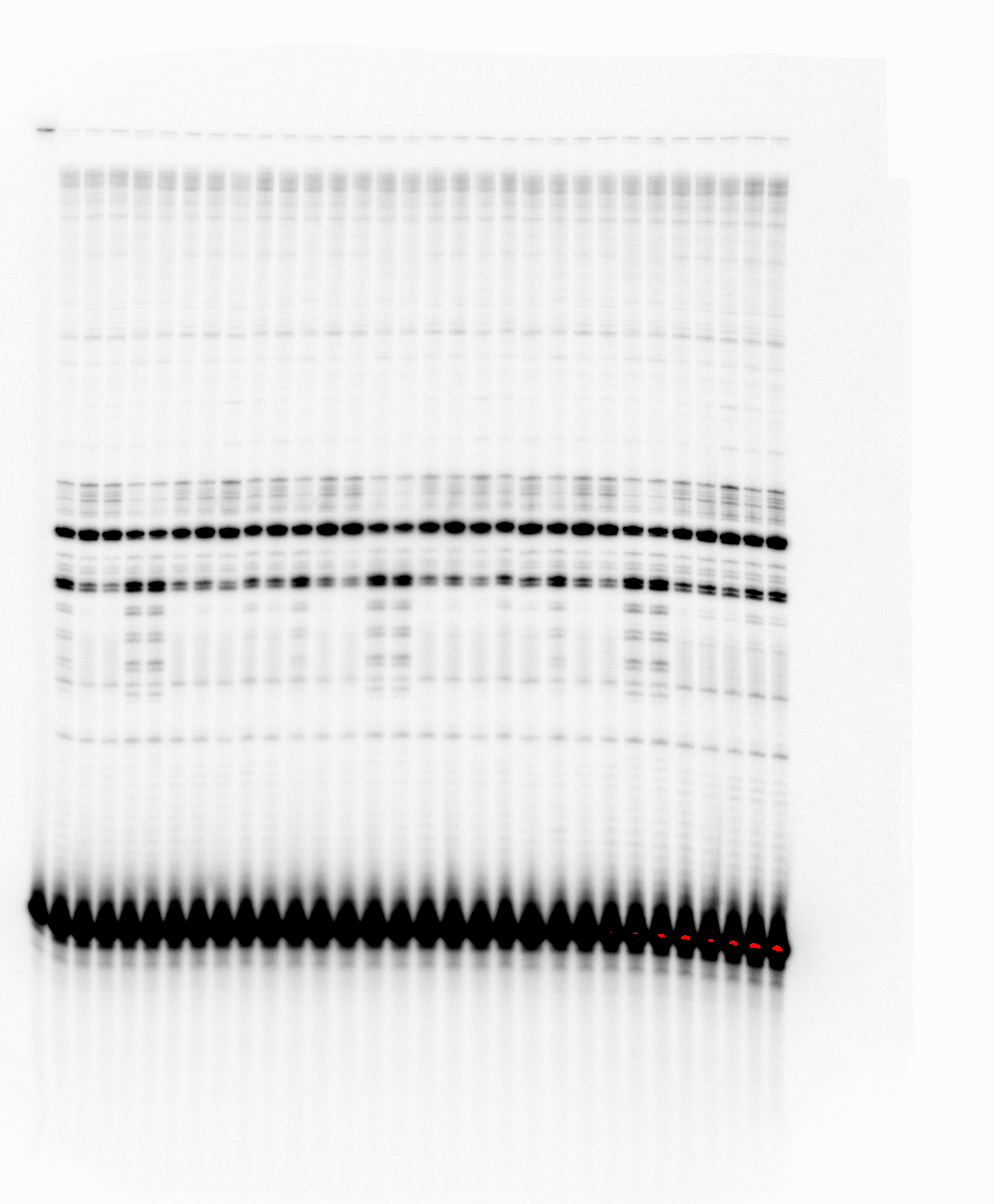

Supplement: Figure 2—figure supplement 2—source data 5. [file elife-71013-fig2-figsupp2-data5.zip › Figure 2-Figure supplement 2-source data 5.tif]

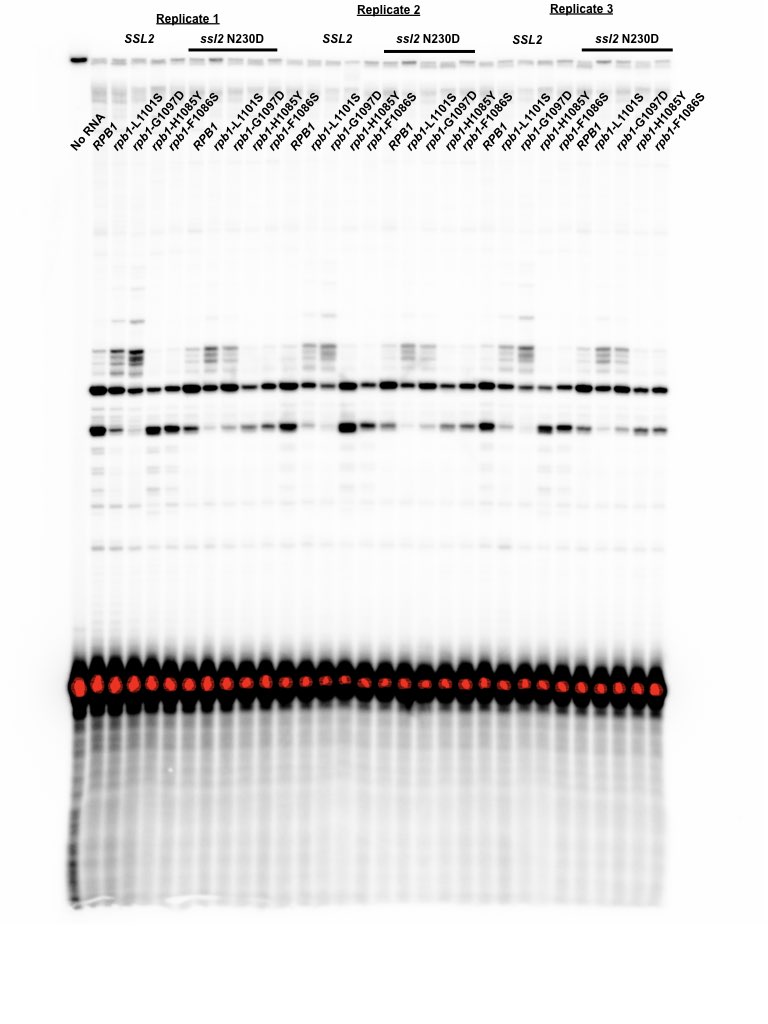

Supplement: Figure 6—source data 2. [file elife-71013-fig6-data2.zip › Figure 6-source data 2.jpeg]

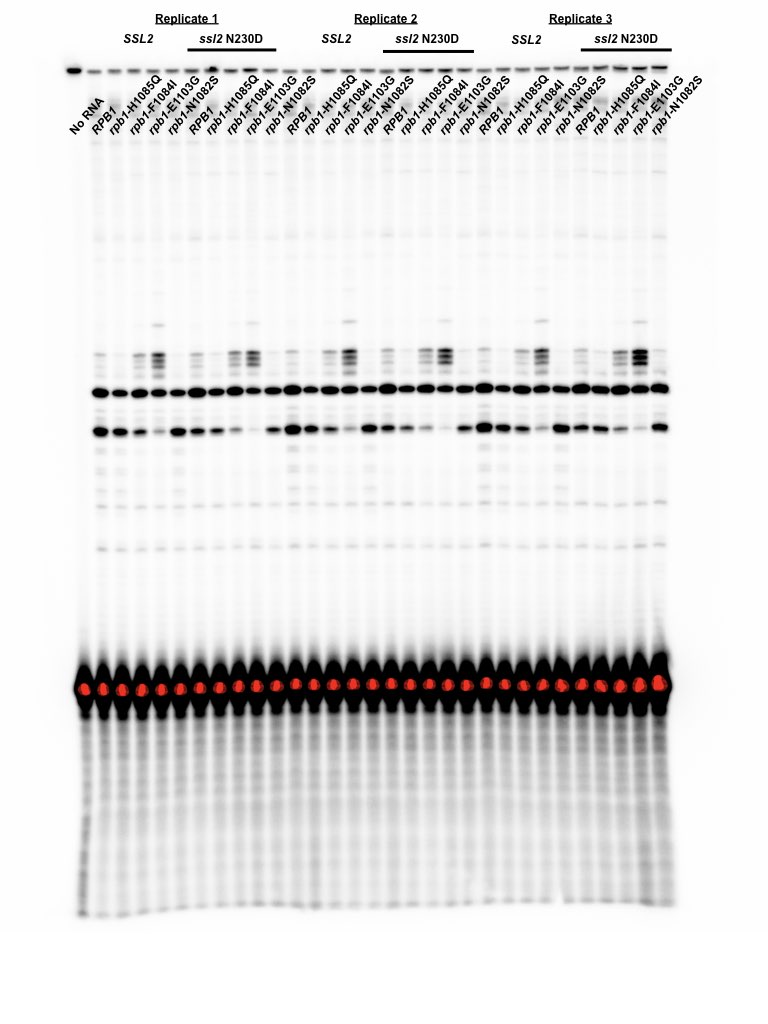

Supplement: Figure 6—source data 3. [file elife-71013-fig6-data3.zip › Figure 6-source data 3.jpeg]

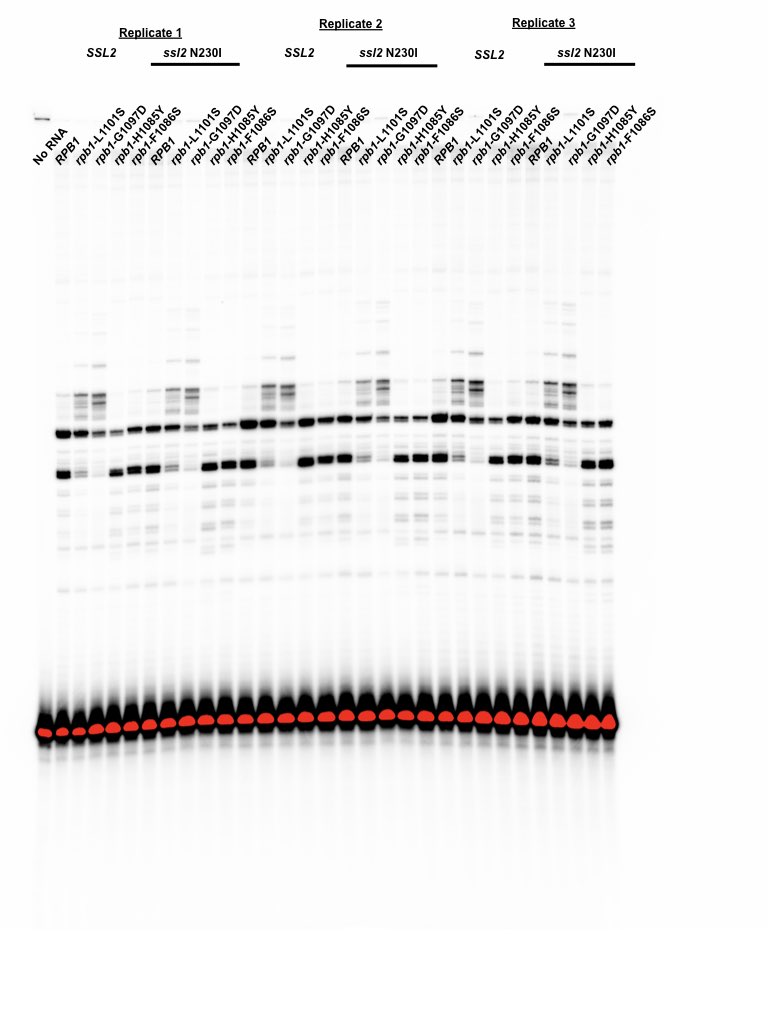

Supplement: Figure 6—source data 7. [file elife-71013-fig6-data7.zip › Figure 6-source data 7.jpeg]

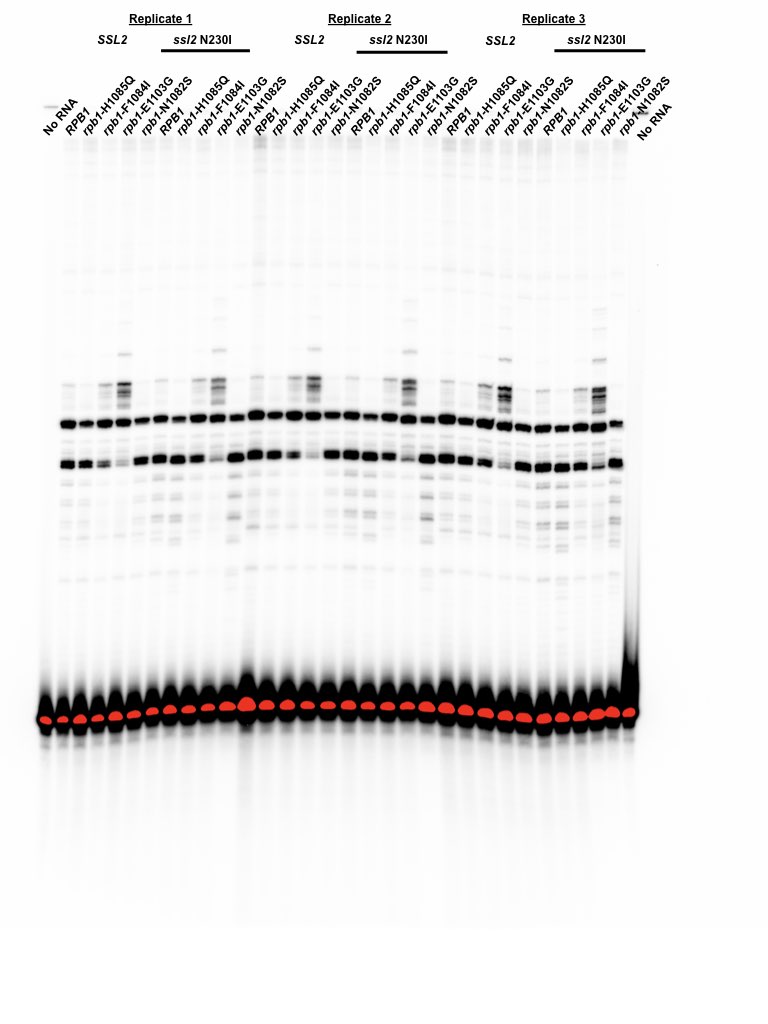

Supplement: Figure 6—source data 8. [file elife-71013-fig6-data8.zip › Figure 6-source data 8.jpeg]

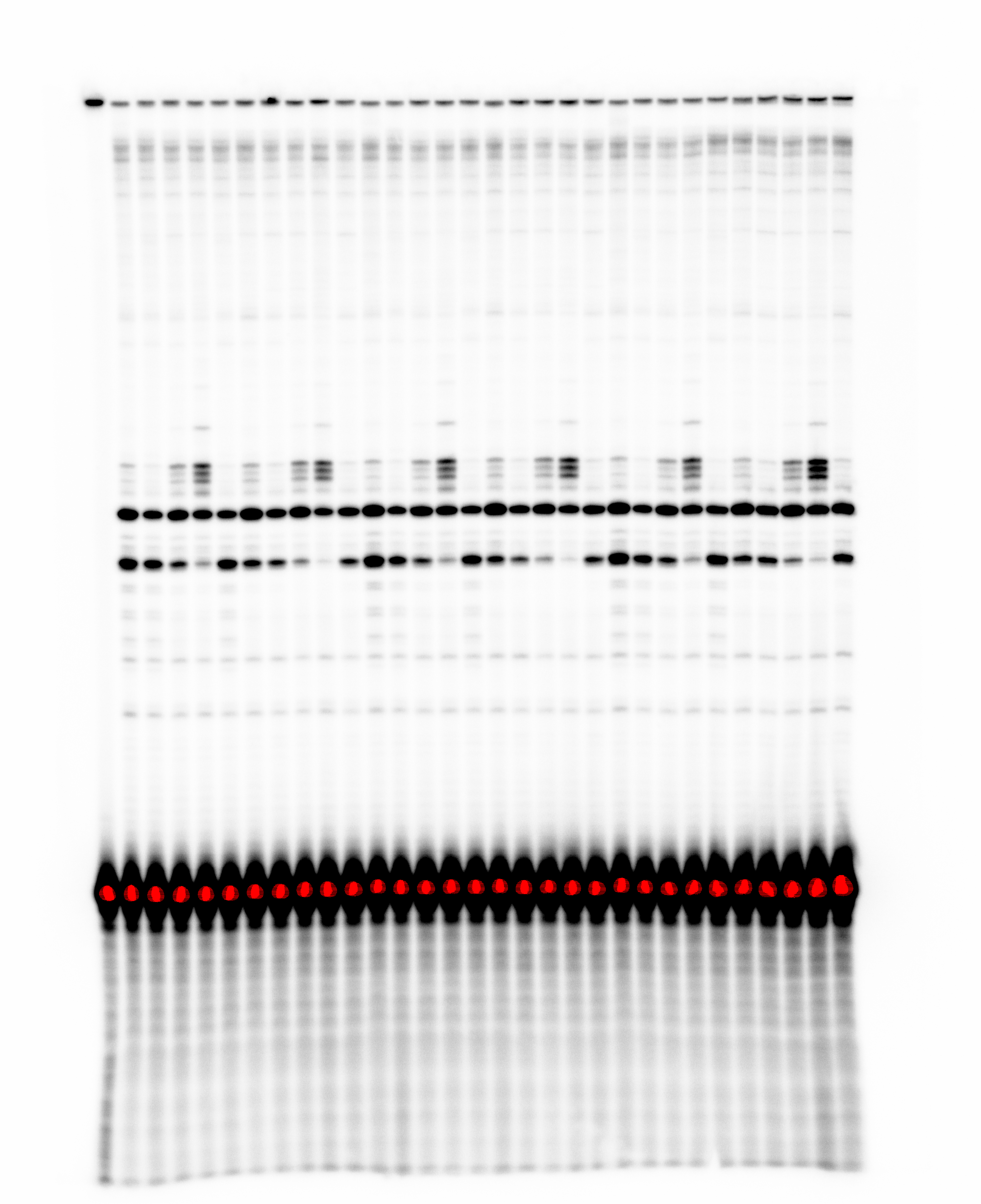

Supplement: Figure 6—source data 13. [file elife-71013-fig6-data13.zip › Figure 6-source data 13.tif]

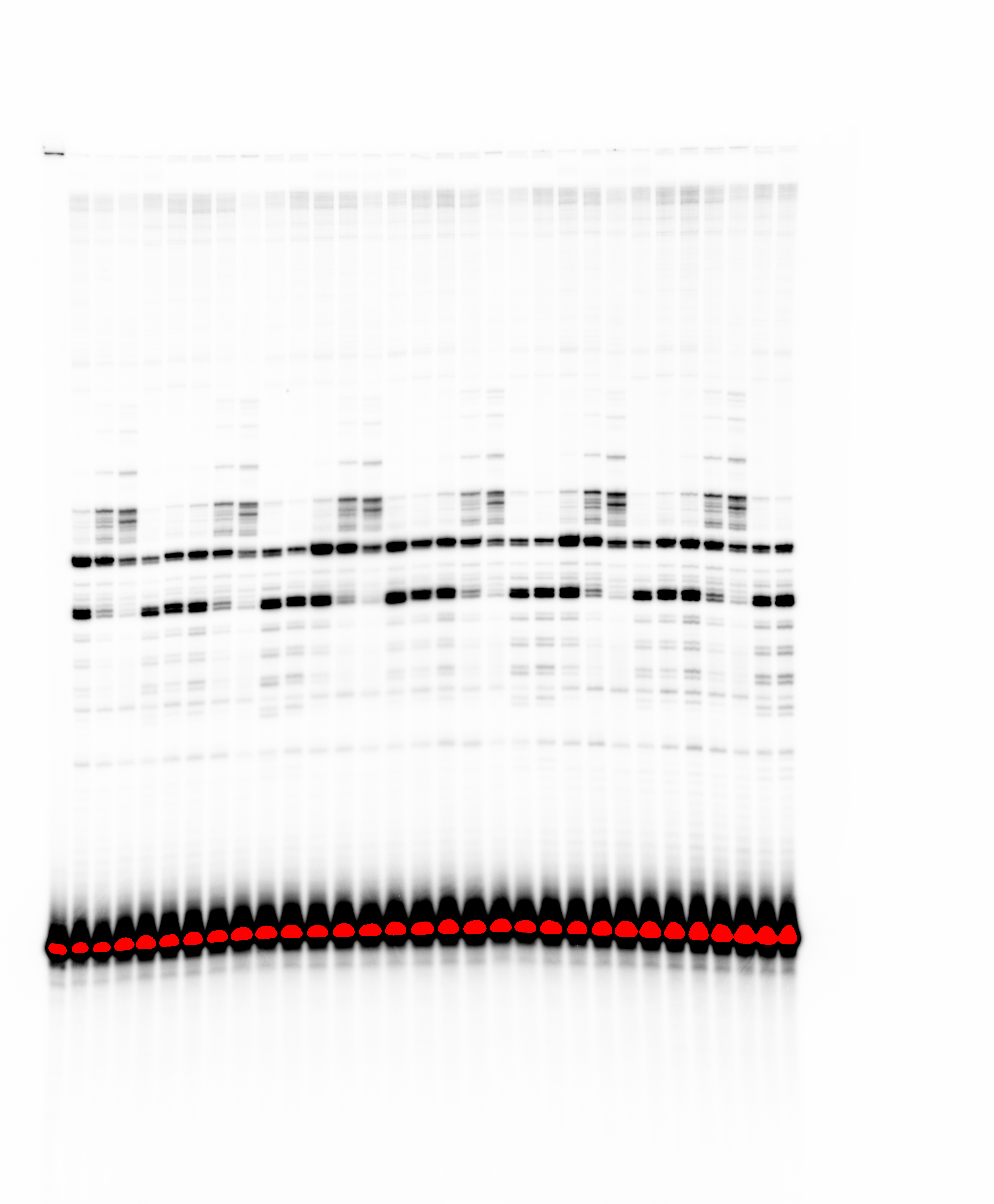

Supplement: Figure 6—source data 14. [file elife-71013-fig6-data14.zip › Figure 6-source data 14.tif]

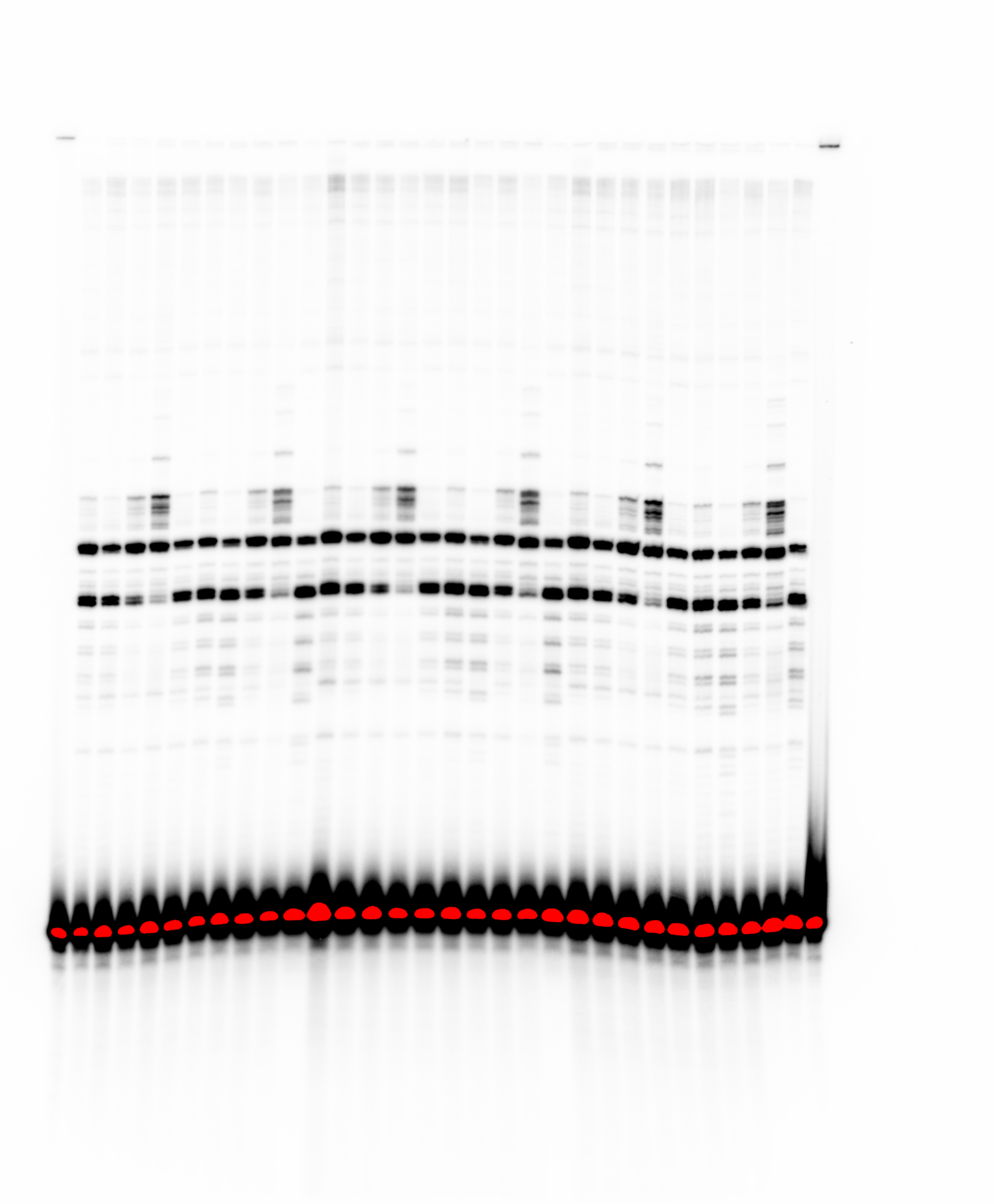

Supplement: Figure 6—source data 15. [file elife-71013-fig6-data15.zip › Figure 6-source data 15.tif]

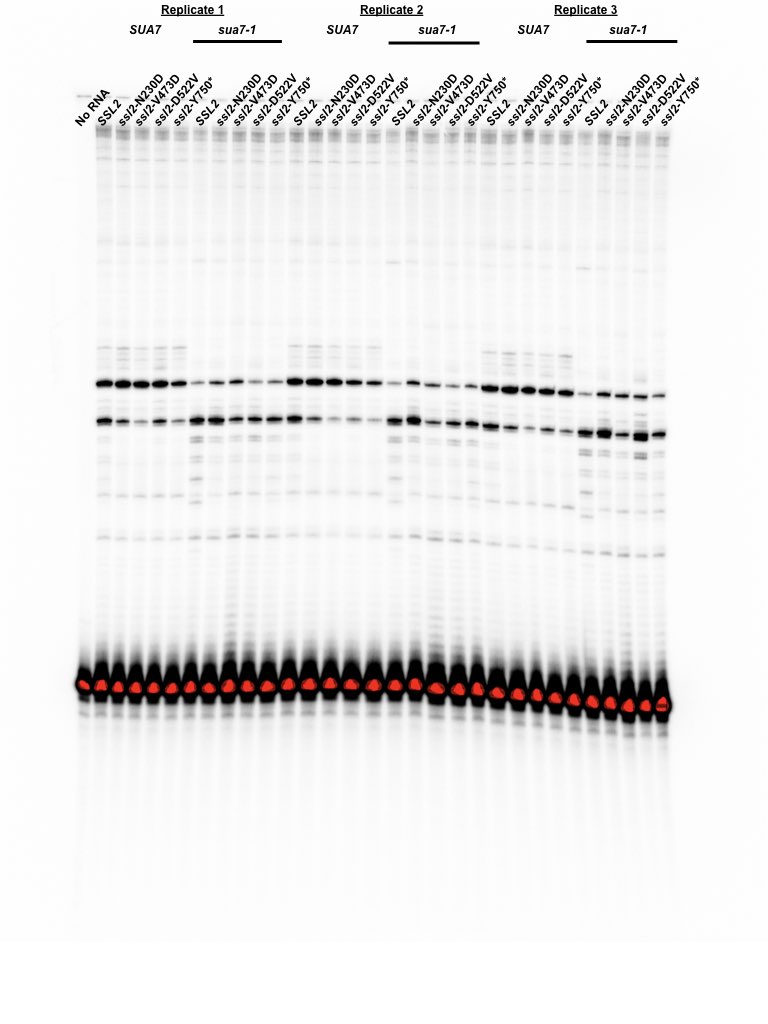

Supplement: Figure 7—figure supplement 1—source data 1. [file elife-71013-fig7-figsupp1-data1.zip › Figure 7-Figure supplement 1-source data 1.jpeg]

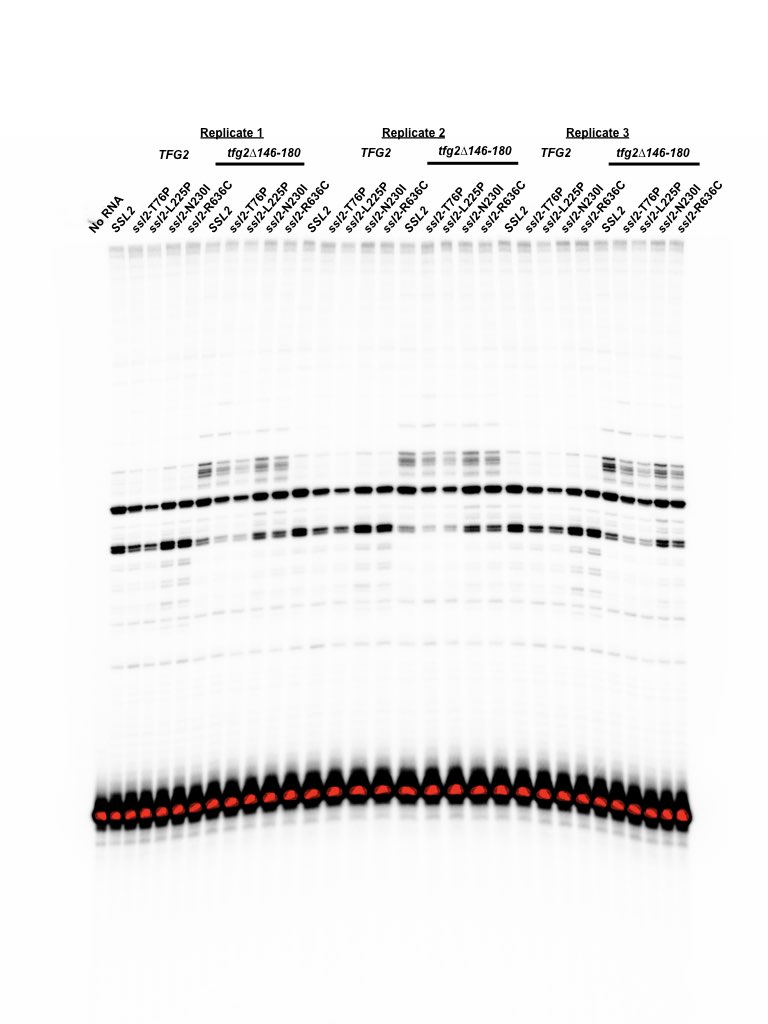

Supplement: Figure 7—figure supplement 1—source data 3. [file elife-71013-fig7-figsupp1-data3.zip › Figure 7-Figure supplement 1-source data 3.jpeg]

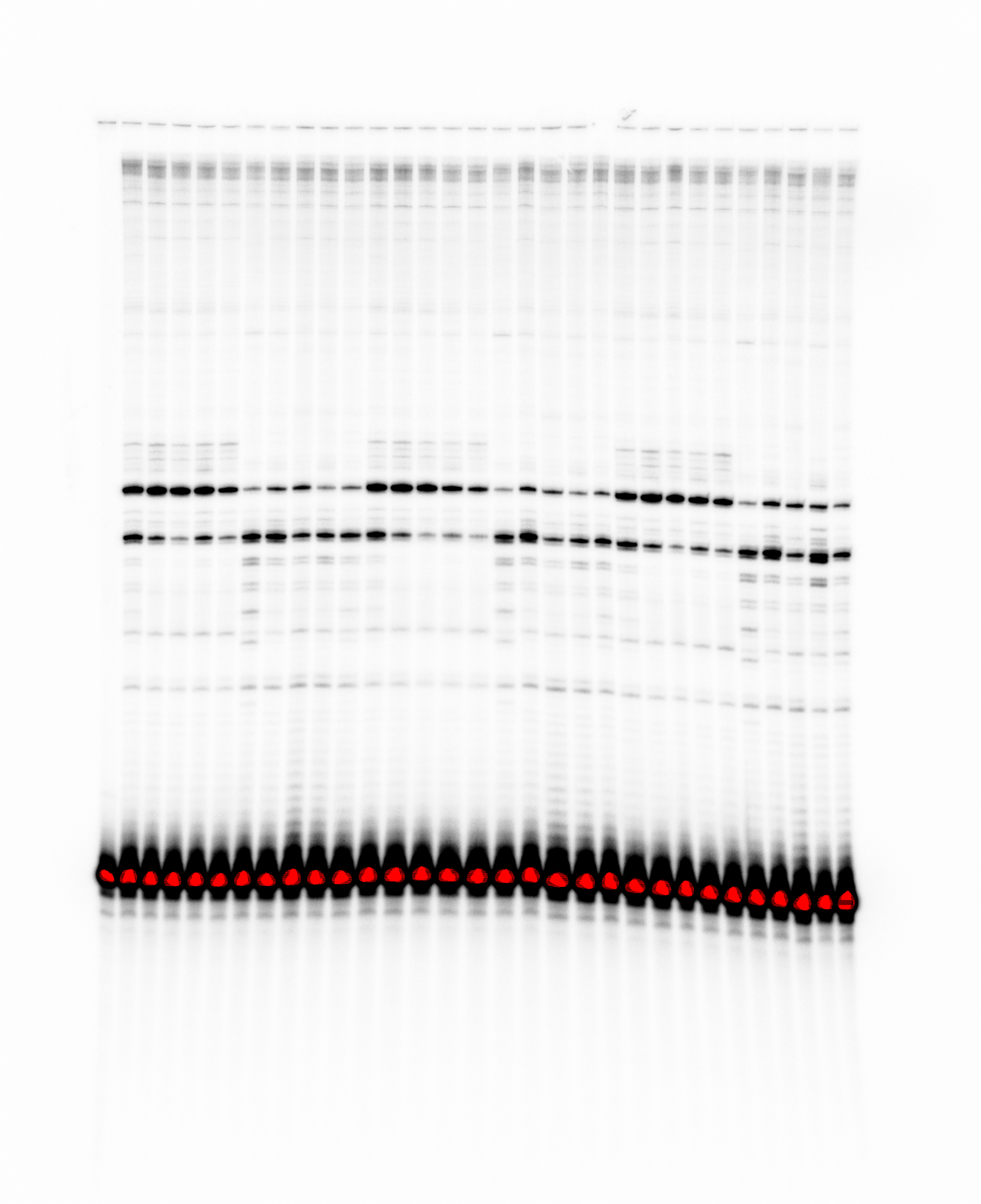

Supplement: Figure 7—figure supplement 1—source data 5. [file elife-71013-fig7-figsupp1-data5.zip › Figure 7-Figure supplement 1-source data 5.tif]

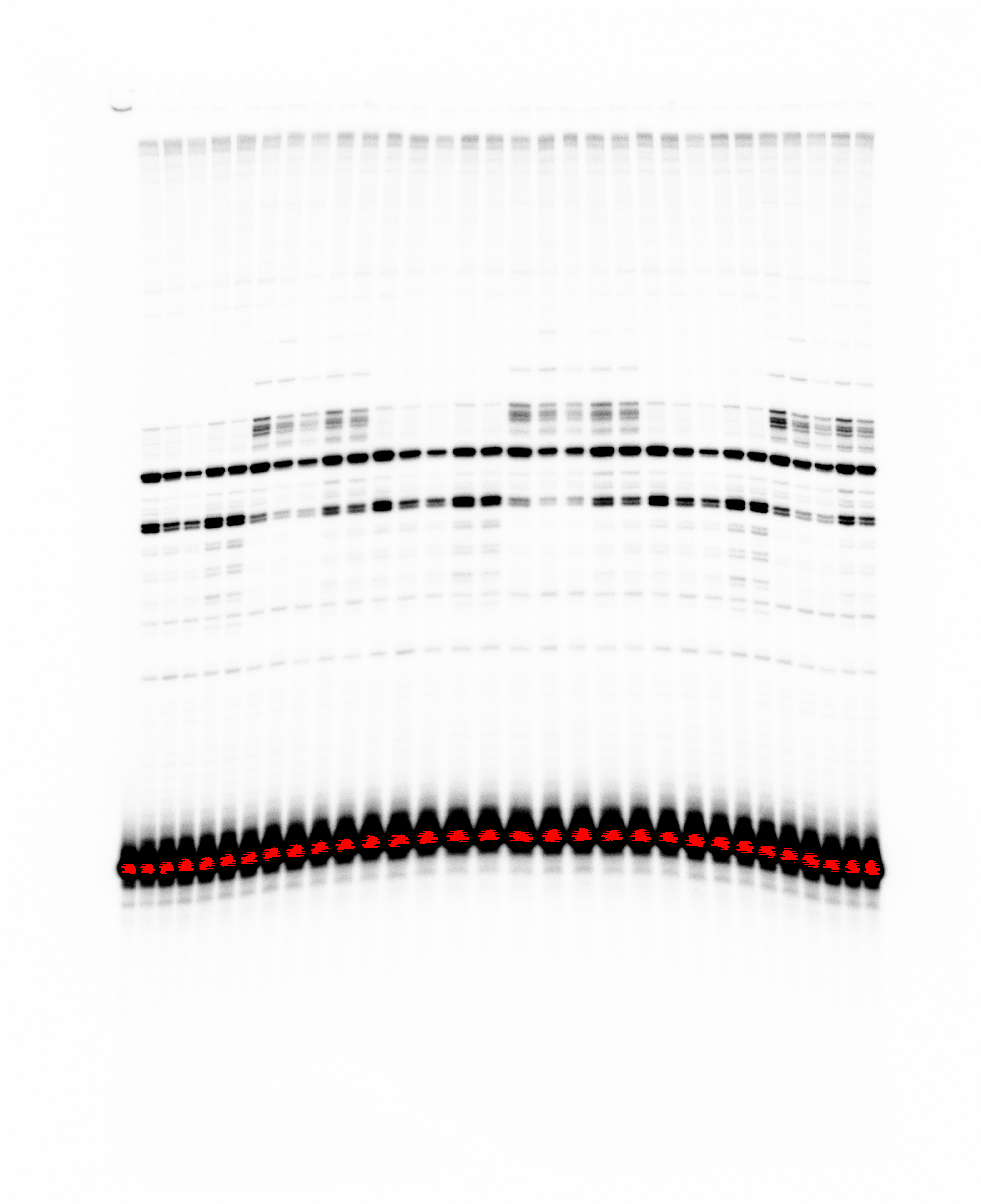

Supplement: Figure 7—figure supplement 1—source data 6. [file elife-71013-fig7-figsupp1-data6.zip › Figure 7-Figure supplement 1-source data 6.tif]

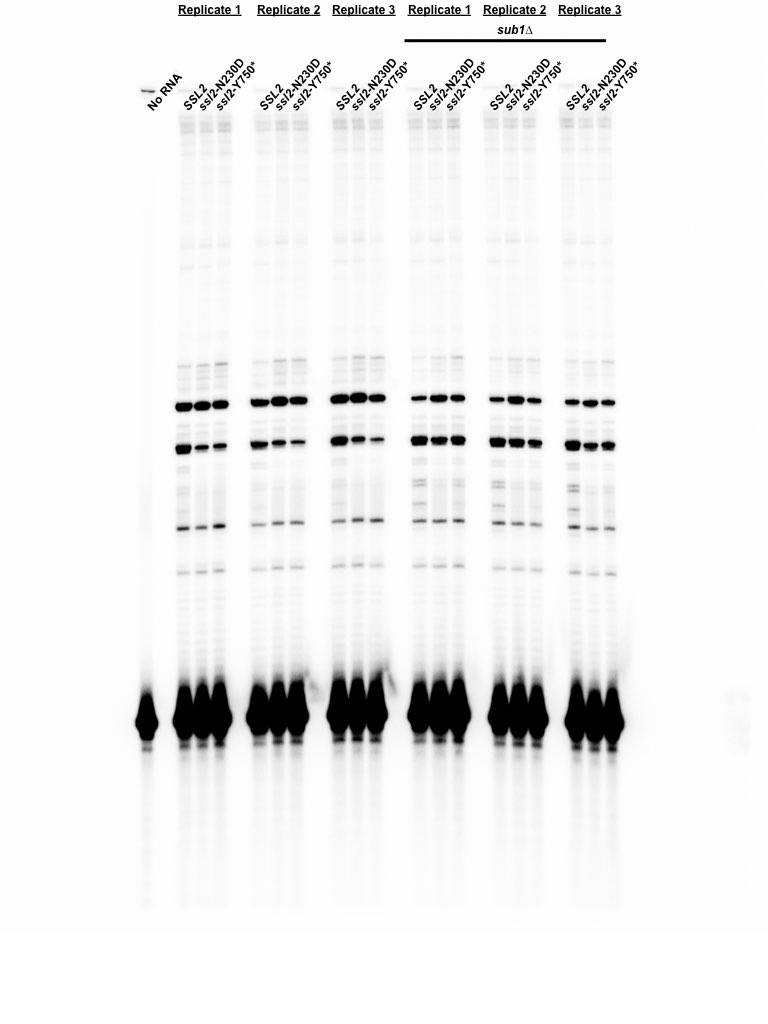

Supplement: Figure 7—figure supplement 2—source data 1. [file elife-71013-fig7-figsupp2-data1.zip › Figure 7-Figure supplement 2-source data 1.jpeg]

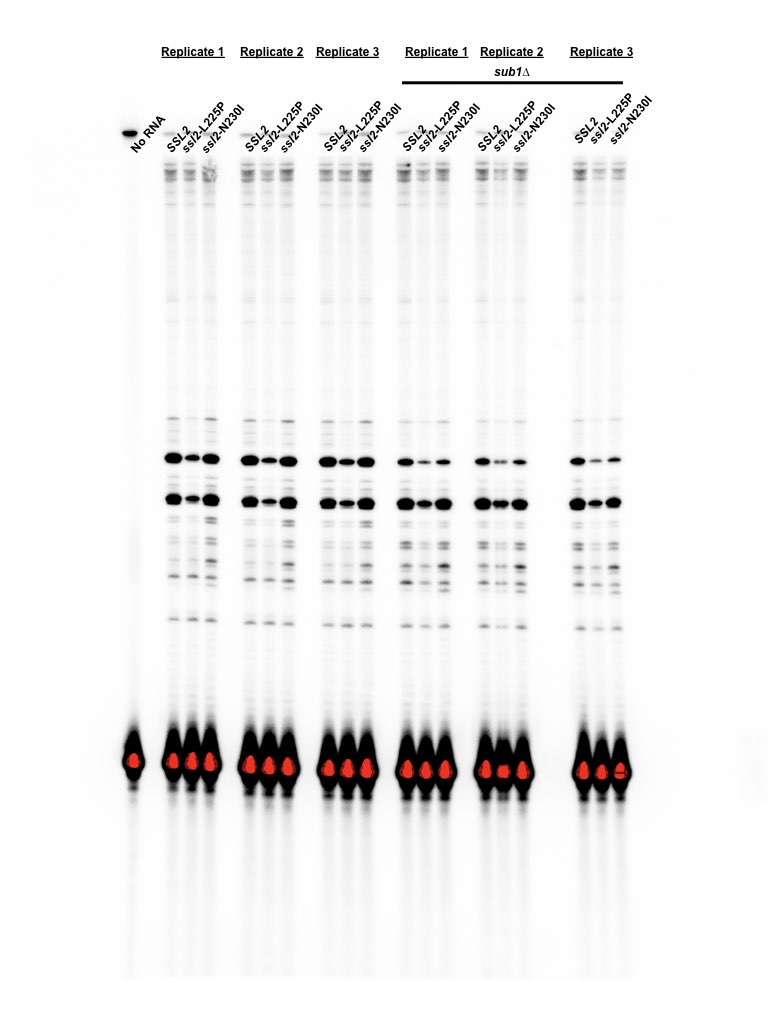

Supplement: Figure 7—figure supplement 2—source data 2. [file elife-71013-fig7-figsupp2-data2.zip › Figure 7-Figure supplement 2-source data 2.jpeg]

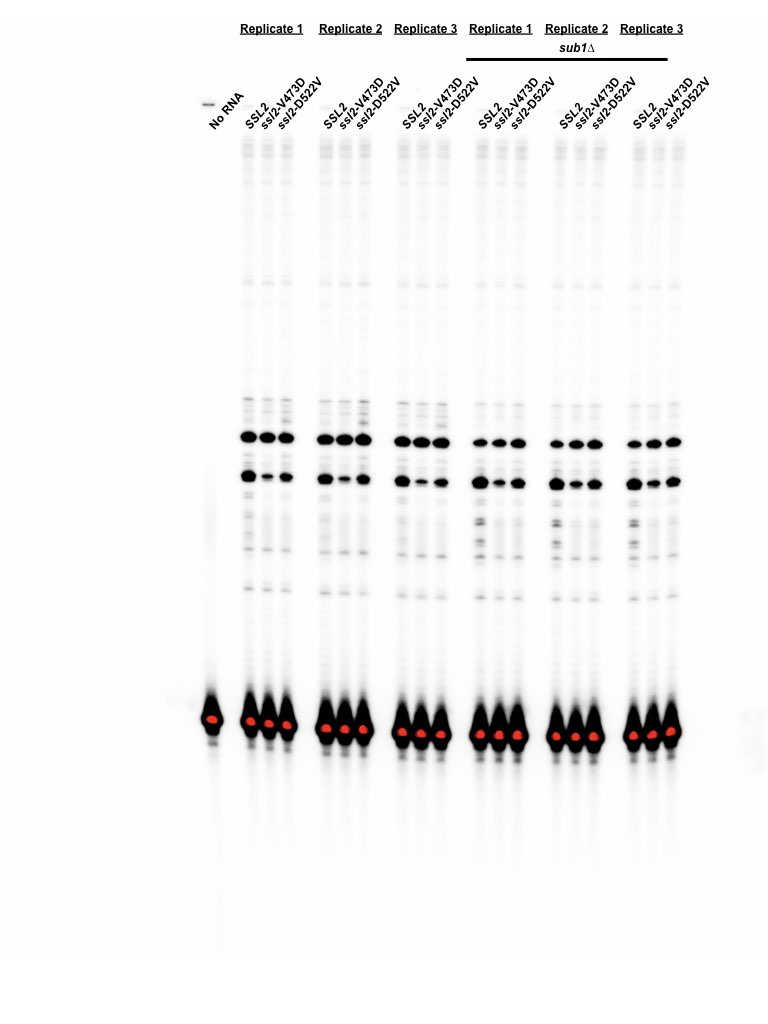

Supplement: Figure 7—figure supplement 2—source data 3. [file elife-71013-fig7-figsupp2-data3.zip › Figure 7-Figure supplement 2-source data 3.jpeg]

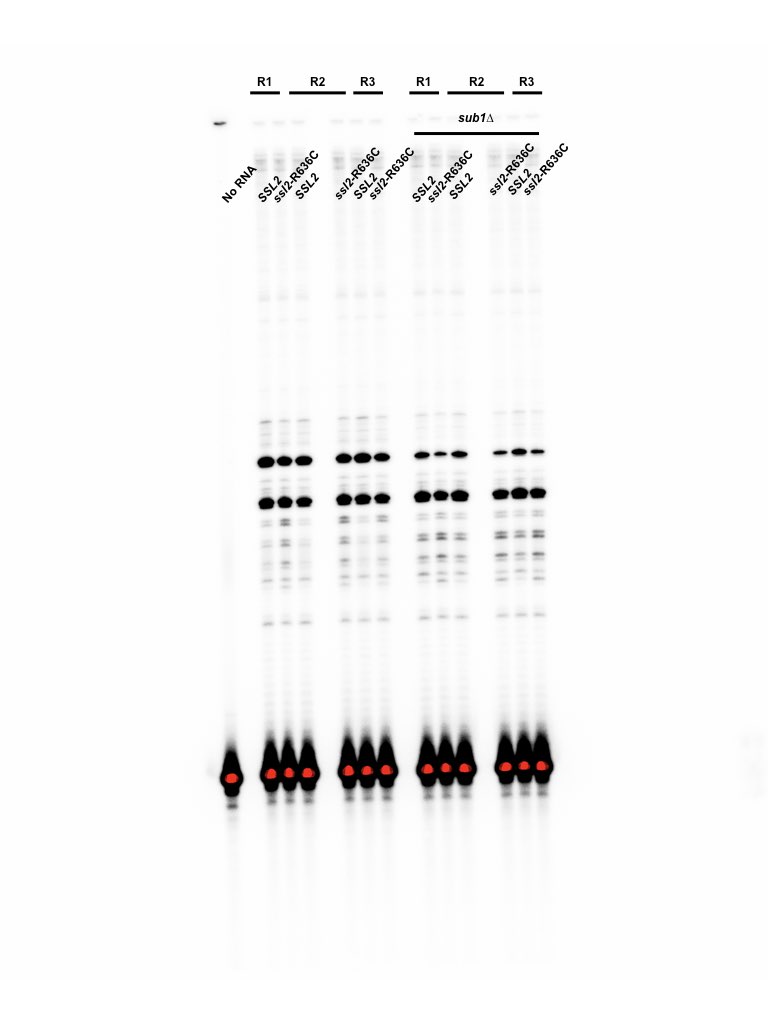

Supplement: Figure 7—figure supplement 2—source data 4. [file elife-71013-fig7-figsupp2-data4.zip › Figure 7-Figure supplement 2-source data 4.jpeg]

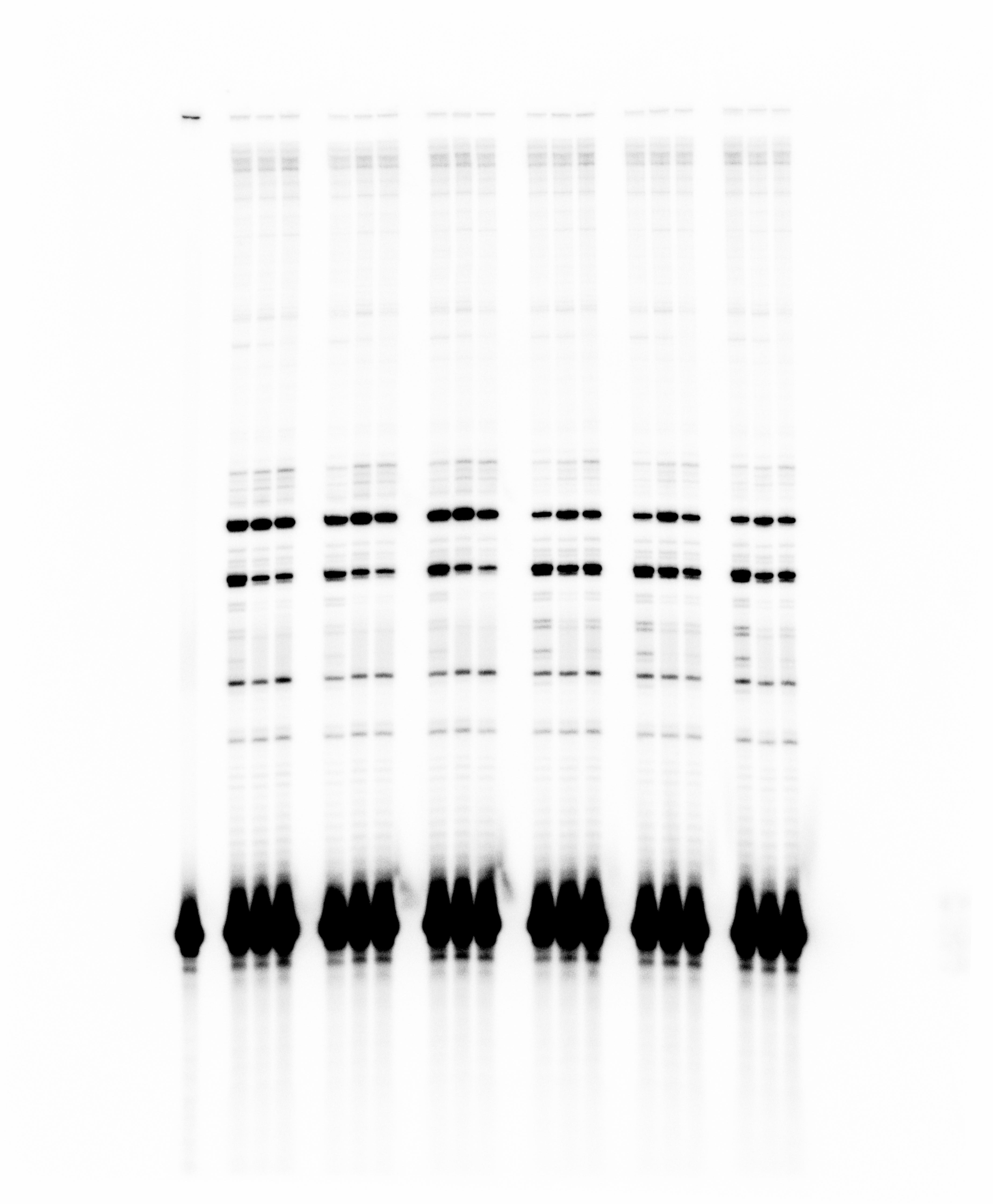

Supplement: Figure 7—figure supplement 2—source data 6. [file elife-71013-fig7-figsupp2-data6.zip › Figure 7-Figure supplement 2-source data 6.tif]

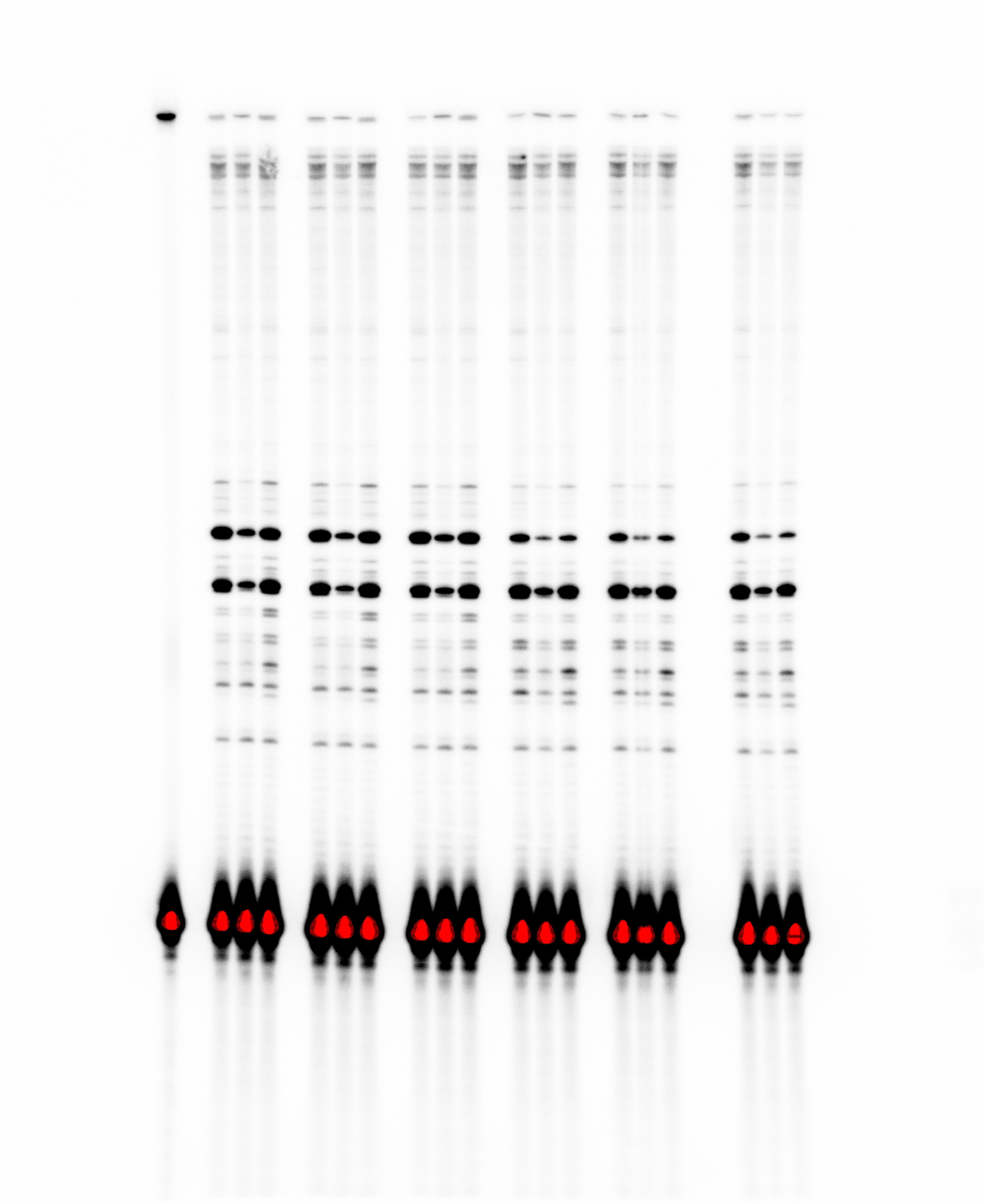

Supplement: Figure 7—figure supplement 2—source data 7. [file elife-71013-fig7-figsupp2-data7.zip › Figure 7-Figure supplement 2-source data 7.tif]

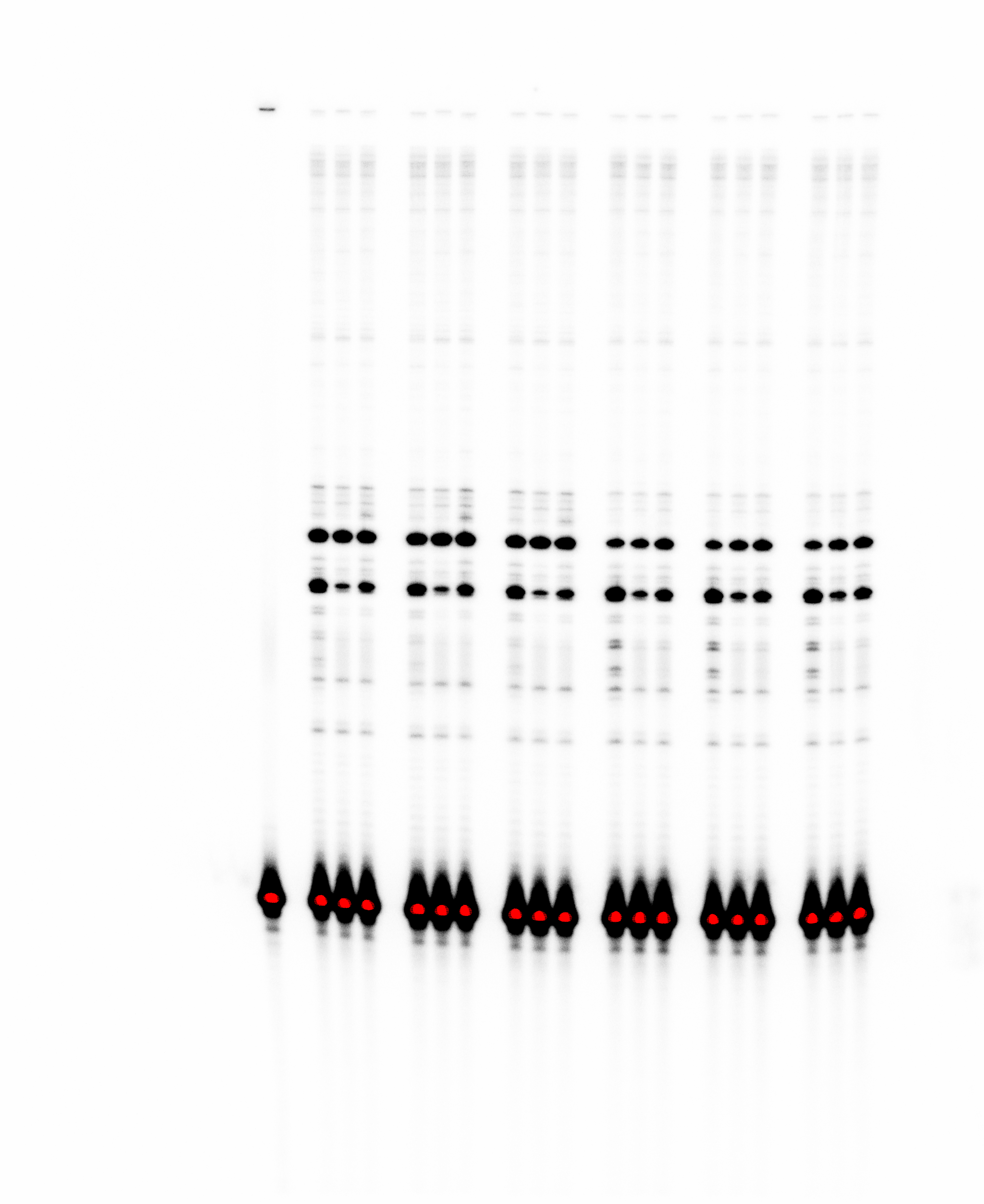

Supplement: Figure 7—figure supplement 2—source data 8. [file elife-71013-fig7-figsupp2-data8.zip › Figure 7-Figure supplement 2-source data 8.tif]

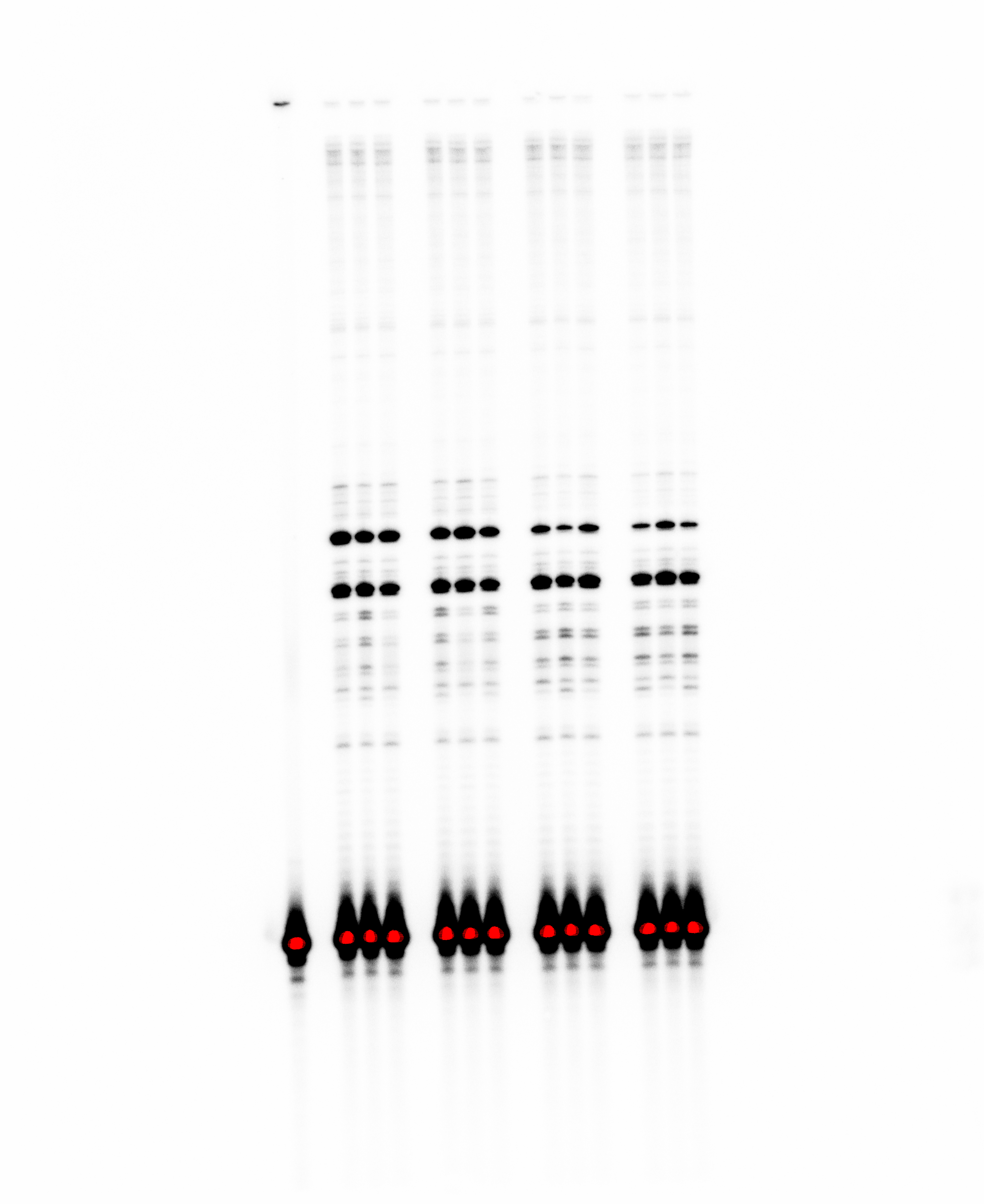

Supplement: Figure 7—figure supplement 2—source data 9. [file elife-71013-fig7-figsupp2-data9.zip › Figure 7-Figure supplement 2-source data 9.tif]
