# Supplementary material for: Ssl2/TFIIH function in transcription start site scanning by RNA polymerase II in Saccharomyces cerevisiae
Source: eLife. 2021 Oct 15;10:e71013. doi: 10.7554/eLife.71013 (PMC8589449; doi:10.7554/eLife.71013)
Supplement: Supplementary file 3. [file elife-71013-supp3.docx]

**Supplementary Table 3 – Source Data Files**

| **Relevant Figure Panel** | **Source Data Type** | **Source data file** |
| --- | --- | --- |
| Figure 1E | Primer Extension Gel(s) | **Figure 1-source data 1** |
| Figure 1G | Primer Extension Gel(s) | **Figure 1-source data 2** |
| Figure 1E,G | Graph data | **Figure 1-source data 3** |
| Figure 1-Figure supplement 1A | Graph data | **Figure 1-Figure supplement 1 source data 1** |
| Figure 2F,H | Graph data | **Figure 2-source data 1** |
| Figure 2-Figure supplement 2A | Primer Extension Gel(s) | **Figure 2-Figure supplement 2-source data 1,2** |
| Figure 2-Figure supplement 2B | Graph data | **Figure 2-Figure supplement 2-source data 3** |
| Figure 3B | Heat scatter data | **GEO GSE182792** |
| Figure 3C | Heat map data | **Figure 3-source data 1** |
| Figure 3-Figure supplement 1A | Heat scatter | **GEO GSE182792** |
| Figure 3-Figure supplement 1B | Heat map data | **Figure 3-Figure supplement 1-source data 1** |
| Figure 4B | ssl2 N230D Heat map data | **Figure 4-source data 1** |
| Figure 4B | ssl2 N230I Heat map data | **Figure 4-source data 2** |
| Figure 4DE | Taf1-Enriched Heat map and graph data | **Figure 4-source data 3** |
| Figure 4DE | Taf1-Depleted Heat map and graph data | **Figure 4-source data 4** |
| Figure 4-Figure supplement 1 | rpb1 E1103G Heat map data | **Figure 4-Figure supplement 1-source data 1** |
| Figure 4-Figure supplement 1 | rpb1 H1085Y Heat map data | **Figure 4-Figure supplement 1-source data 2** |
| Figure 4-Figure supplement 1 | ssl2 L225P Heat map data | **Figure 4-Figure supplement 1-source data 3** |
| Figure 4-Figure supplement 1 | ssl2 D522V Heat map data | **Figure 4-Figure supplement 1-source data 4** |
| Figure 4-Figure supplement 1 | ssl2 R636C Heat map data | **Figure 4-Figure supplement 1-source data 5** |
| Figure 4-Figure supplement 1 | ssl2 Y750* Heat map data | **Figure 4-Figure supplement 1-source data 6** |
| Figure 4-Figure supplement 2A | Heat map data | **Figure 4-Figure supplement 2-source data 1** |
| Figure 5B | Taf1-Enriched Graph data | **Figure 5-source data 1** |
| Figure 5B | Taf1-Depleted Graph data | **Figure 5-source data 2** |
| Figure 5CD | Taf1-Enriched Heat map and graph data | **Figure 5-source data 3** |
| Figure 5CD | Taf1-Depleted Heat map and graph data | **Figure 5-source data 4** |
| Figure 5-Figure supplement 1A | Heat map data | **Figure 5-Figure supplement 1-source data 1** |
| Figure 5-Figure supplement 2A | Graph data | **Figure 5-Figure supplement 2-source data 1** |
| Figure 5-Figure supplement 2B | Data table | **Figure 5-Figure supplement 2-source data 2** |
| Figure 5-Figure supplement 2B | Graph data | **Figure 5-Figure supplement 2-source data 3** |
| Figure 5-Figure supplement 2C | Data table | **Figure 5-Figure supplement 2-source data 4** |
| Figure 5-Figure supplement 2C | Graph data | **Figure 5-Figure supplement 2-source data 5** |
| Figure 5-Figure supplement 2D | Graph data | **Figure 5-Figure supplement 2-source data 6** |
| Figure 5-Figure supplement 2E | Data table | **Figure 5-Figure supplement 2-source data 7** |
| Figure 5-Figure supplement 2E | Graph data | **Figure 5-Figure supplement 2-source data 8** |
| Figure 5-Figure supplement 2F | Data table | **Figure 5-Figure supplement 2-source data 9** |
| Figure 5-Figure supplement 2F | Graph data | **Figure 5-Figure supplement 2-source data 10** |
| Figure 6G | Heat map data | **Figure 6-source data 1** |
| Figure 6H | Primer extension gel | **Figure 6-source data 2** |
| Figure 6H | Primer extension gel | **Figure 6-source data 3** |
| Figure 6I | rpb1 singles Heat map data | **Figure 6-source data 4** |
| Figure 6I | N230D single Heat map data | **Figure 6-source data 5** |
| Figure 6I | N230D double Heat map data | **Figure 6-source data 6** |
| Figure 6J | Primer extension gel | **Figure 6-source data 7** |
| Figure 6J | Primer extension gel | **Figure 6-source data 8** |
| Figure 6K | rpb1 singles Heat map data | **Figure 6-source data 9** |
| Figure 6K | N230I single Heat map data | **Figure 6-source data 10** |
| Figure 6K | N230I double Heat map data | **Figure 6-source data 11** |
| Figure 6-Figure supplement 3A | Graph data | **Figure 6-Figure supplement 3-source data 1** |
| Figure 6-Figure supplement 3B | Graph data | **Figure 6-Figure supplement 3-source data 2** |
| Figure 7A | Heat map data | **Figure 7-source data 1** |
| Figure 7B | Heat map data | **Figure 7-source data 2** |
| Figure 7C | Heat map data | **Figure 7-source data 3** |
| Figure 7D | Heat map data | **Figure 7-source data 4** |
| Figure 7-Figure supplement 1C | Primer extension gel | **Figure 7-Figure supplement 1-source data 1** |
| Figure 7-Figure supplement 1D | Graph data | **Figure 7-Figure supplement 1-source data 2** |
| Figure 7-Figure supplement 1G | Primer extension gel | **Figure 7-Figure supplement 1-source data 3** |
| Figure 7-Figure supplement 1H | Graph data | **Figure 7-Figure supplement 1-source data 4** |
| Figure 7-Figure supplement 2B | Primer extension gel | **Figure 7-Figure supplement 2-source data 1** |
| Figure 7-Figure supplement 2B | Primer extension gel | **Figure 7-Figure supplement 2-source data 2** |
| Figure 7-Figure supplement 2B | Primer extension gel | **Figure 7-Figure supplement 2-source data 3** |
| Figure 7-Figure supplement 2B | Primer extension gel | **Figure 7-Figure supplement 2-source data 4** |
| Figure 7-Figure supplement 2C | Graph data | **Figure 7-Figure supplement 2-source data 5** |
| Figure 9-Figure supplement 1A | Graph data | **Figure 9-Figure supplement 1-source data 1** |
| Figure 9-Figure supplement 1B | Graph data | **Figure 9-Figure supplement 1-source data 2** |

Raw gels files

| **Relevant Figure Panel** | **Source Data Type** | **Source data file** | **Annotated source data file** |
| --- | --- | --- | --- |
| Figure 1E | Primer Extension Gel(s) | **Figure 1-source data 4** | **Figure 1-source data 1** |
| Figure 1G | Primer Extension Gel(s) | **Figure 1-source data 5** | **Figure 1-source data 2** |
| Figure 2-Figure supplement 2A | Primer Extension Gel(s) | **Figure 2-Figure supplement 2-source data 4,5** | **Figure 2-Figure supplement 2-source data 1,2** |
| Figure 6H | Primer extension gel | **Figure 6-source data 12** | **Figure 6-source data 2** |
| Figure 6H | Primer extension gel | **Figure 6-source data 13** | **Figure 6-source data 3** |
| Figure 6J | Primer extension gel | **Figure 6-source data 14** | **Figure 6-source data 7** |
| Figure 6J | Primer extension gel | **Figure 6-source data 15** | **Figure 6-source data 8** |
| Figure 7-Figure supplement 1C | Primer extension gel | **Figure 7-Figure supplement 1-source data 5** | **Figure 7-Figure supplement 1-source data 1** |
| Figure 7-Figure supplement 1G | Primer extension gel | **Figure 7-Figure supplement 1-source data 6** | **Figure 7-Figure supplement 1-source data 3** |
| Figure 7-Figure supplement 2B | Primer extension gel | **Figure 7-Figure supplement 2-source data 6** | **Figure 7-Figure supplement 2-source data 1** |
| Figure 7-Figure supplement 2B | Primer extension gel | **Figure 7-Figure supplement 2-source data 7** | **Figure 7-Figure supplement 2-source data 2** |
| Figure 7-Figure supplement 2B | Primer extension gel | **Figure 7-Figure supplement 2-source data 8** | **Figure 7-Figure supplement 2-source data 3** |
| Figure 7-Figure supplement 2B | Primer extension gel | **Figure 7-Figure supplement 2-source data 9** | **Figure 7-Figure supplement 2-source data 4** |
